# Emergency department crowding increases 10-day mortality for non-critical patients: a retrospective observational study

**DOI:** 10.1007/s11739-023-03392-8

**Published:** 2023-08-22

**Authors:** Anna Eidstø, Jari Ylä-Mattila, Jalmari Tuominen, Heini Huhtala, Ari Palomäki, Teemu Koivistoinen

**Affiliations:** 1 Emergency Department, Tampere University Hospital, P.O. Box 2000, 33521 Tampere, Finland; 2 Faculty of Medicine and Health Technology, Tampere University, 33014 Tampere, Finland; 3 Faculty of Social Sciences, Tampere University, 33014 Tampere, Finland; 4 Emergency Department, Kanta-Häme Central Hospital, 13530 Hämeenlinna, Finland

**Keywords:** Adverse patient outcome, Crowding, Emergency Department, Mortality, Emergency Department occupancy ratio

## Abstract

The current evidence suggests that higher levels of crowding in the Emergency Department (ED) have a negative impact on patient outcomes, including mortality. However, only limited data are available about the association between crowding and mortality, especially for patients discharged from the ED. The primary objective of this study was to establish the association between ED crowding and overall 10-day mortality for non-critical patients. The secondary objective was to perform a subgroup analysis of mortality risk separately for both admitted and discharged patients. An observational single-centre retrospective study was conducted in the Tampere University Hospital ED from January 2018 to February 2020. The ED Occupancy Ratio (EDOR) was used to describe the level of crowding and it was calculated both at patient’s arrival and at the maximum point during the stay in the ED. Age, gender, Emergency Medical Service transport, triage acuity, and shift were considered as confounding factors in the analyses. A total of 103,196 ED visits were included. The overall 10-day mortality rate was 1.0% (*n* = 1022). After controlling for confounding factors, the highest quartile of crowding was identified as an independent risk factor for 10-day mortality. The results were essentially similar whether using the EDOR at arrival (OR 1.31, 95% CI 1.07–1.61, *p* = 0.009) or the maximum EDOR (OR 1.27, 95% CI 1.04–1.56, *p* = 0.020). A more precise, mortality-associated threshold of crowding was identified at EDOR 0.9. The subgroup analysis did not yield any statistically significant findings. The risk for 10-day mortality increased among non-critical ED patients treated during the highest EDOR quartile.

**Supplementary Information:**

The online version contains supplementary material available at 10.1007/s11739-023-03392-8.

## Background

Over the past two decades, Emergency Department (ED) crowding has become a globally recognized issue [1–4]. Prior studies have associated crowding with adverse patient outcomes, including increased mortality, longer in-patient length of stays, higher costs, and more medication errors [5–13]. However, some studies have also found no connection between ED crowding and mortality, or their results have varied between EDs [12–14].

A widely accepted definition for crowding does not exist, and a great variety of different crowding measures have thus been presented previously [15]. In prior mortality studies, suboptimal proxy measures of crowding have often been used, such as daily ambulance diversion hours, patient waiting times, or number of crowded shifts [6, 9, 11, 14]. Two different reviews suggest that the most reliable crowding measures are based on time intervals and patient counts, such as ED length of stay (ED LOS) and ED occupancy ratio (EDOR) [15, 16]. In recent studies there has also been a tendency to favour the EDOR standard (i.e., patients divided by available beds) for each visit, which is not only more generalizable, but also a more specific way to define the effect of crowding on the individual patient [7, 10, 12, 13].

The effects of ED crowding on short-time mortality have been previously studied, but mostly among admitted patients; thus, the data of discharged ED patients is still very limited [6–11, 13]. In addition, some studies have included only patients with health insurance due to lack of available data on those without coverage [12].

The aim of this study was to establish the association between ED crowding and overall, 10-day mortality rates in a Finnish tertiary hospital using the Occupancy Ratio to accurately measure the level of crowding. In addition, we conducted subgroup analyses on both admitted and discharged patients to explore any differences in how crowding affects different patient subgroups.

## Methods

### Study design and setting

A retrospective single-centre observational cohort study was conducted in the ED of Tampere University Hospital, Finland. Tampere University Hospital provides secondary care for more than 500,000 residents in the Pirkanmaa Hospital District, and it is the only hospital in the region that manages all the severe emergency situations. In addition, the hospital is a tertiary care unit for a catchment area of over 900,000 residents. It is one of the largest Emergency Departments in the Nordic European Countries based on annual visits of around 90,000 per year. The treatment of patients under 16 years without an acute traumatic injury was gradually taken over by the hospital’s Paediatric Unit from September of 2018 and through the year 2019, thus, the study population mostly consisted of adults (1472 children < 16 years included).

This ED has a total of 65 beds that are divided into resuscitation, medical and surgical treatment spaces with 6, 36, and 23 beds, respectively. In addition, there is a waiting room for walk-in patients who are not in need of continuous surveillance. Critical patients with marked disturbances in their vital functions are treated in the resuscitation room and prioritized over other patients.

According to Finnish legislation, register studies do not require approval by a hospital ethics committee [17]. However, this study was approved by the hospital’s Research Director (R22601). The STROBE (Strengthening the Reporting of Observational Studies in Epidemiology) guidelines were also applied in this study [18].

### Data sources and variables

Hospital data management services provided the data for all ED visits during the study period selected from 1 January 2018 to 29 February 2020, resulting in a sample of 26 consecutive months. The following variables were collected for each patient visit: personal identity code, gender, date of birth, age at arrival, date and time of arrival and discharge, Emergency Medical Service (EMS) transport, Emergency Severity Index (ESI) classification [19], admission or non-admission to hospital, whether the patient died within 10 days after the ED visit and the date of death. The shift for each visit was defined as the time of arrival (day 8.00–15.59, evening 16.00–22.59, and night 23.00–7.59).

**Fig. 1:**
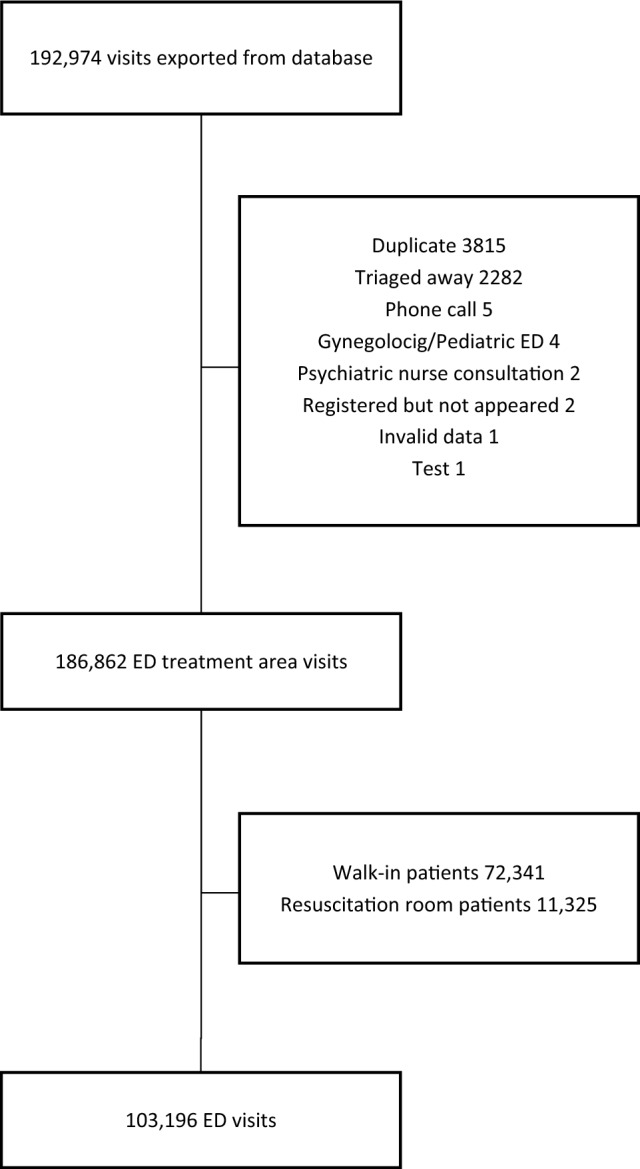
Study selection flow chart

Patients in the ED were divided into four groups, based on the latest treatment space occupied before discharge: medical, surgical, resuscitation room, or walk-in. Preliminary analysis confirmed the hypothesis that the mortality rate was extremely low (0.02%) among walk-in patients and, even though the resuscitation room is always prioritized regardless of crowding, the mortality rate among the critical patients was as high as 9.8%. These groups were excluded from the final study cohort (Fig. 1).

Other excluded data included phone calls, patients diverted by the triage, those confirmed dead upon arrival, duplicates, and four patients who had been accidentally admitted to ED, but were immediately transferred to the Gynaecologic or Paediatric Unit without any interventions in the ED. One entry was excluded because of incomplete data. Furthermore, if there was more than one visit within 10 days prior to a patient’s death, only the most recent visit was chosen for observation.

Data on ESI scores were missing in 207 cases, and EMS transport information was missing in 31 cases; as a result, these cases were excluded from the logistic regression analyses.

Temporal occupancy was calculated based on registered arrival and discharge times. The EDOR for each visit was counted separately for the different treatment spaces (medical and surgical), since these hospital spaces have their own staffing resources. These groups were analysed as a combined group. Two different EDOR metrics were included: EDOR_A_ denoting occupancy at arrival and EDOR_MAX_ denoting the highest recorded occupancy for the ED unit during the visit.

### Statistical analyses

Continuous data were presented as the mean and standard deviation (SD) or as the median and interquartile range (IQR). Nominal and categorical data were presented as numbers and percentages. Logistic regression analyses results were presented as odds ratios (OR) with 95% confidence intervals (95% CI). Statistical significance was tested using the chi-squared test, and *p* values < 0.05 were considered as being statistically significant.

The analyses were performed using both crowding metrics: occupancy at arrival (EDOR_A_) and the highest occupancy (EDOR_MAX_). The EDORs were divided and analysed first in quartiles. Patients who visited the ED during the least crowded quartile (Q1) were considered the reference group for the other quartiles (Q2–4), to determine, whether the crowding status affects patient mortality rates. In further analysis the EDORs were divided in 10 deciles to determine the actual threshold value for increased mortality.

The multivariable logistic regression analyses were adjusted for age, sex, shift, ESI score, and transport mode to the ED (EMS or other), ESI, and transport mode indicating the severity of illness. ESI scores were reduced to three variables (ESI 1–2, ESI 3, and ESI 4–5) because of the relatively small number of ESI 1 and ESI 5 patients.

All statistical analyses were performed using IBM SPSS Statistics (Version: 28.0.1.1).

## Results

The final study cohort consisted of 103,196 visits from 61,297 individuals. The total hospital admission rate was 56% (*n* = 57,746). There were 1022 (1.0%) deaths within 10 days, 9.8% (*n* = 100) of these were patients discharged from the ED and 90.2% (*n* = 922) had been admitted to the hospital. The median time from ED visit to death was 5.0 (IQR: 3.0–8.0) days. These patient characteristics are shown in Table 1, where the crowding status is defined by quartiles of EDOR at arrival (EDOR_A_).

**Table 1:** Characteristics of included Emergency Department patients, crowding status defined at arrival (EDOR_A_)

	Total		EDOR at patient’s arrival							
			Q1 (< 0.36)		Q2 (0.36–0.54)		Q3 (0.54–0.75)		Q4 (> 0.75)	
	*n* = 103,196		*n* = 23,862		*n* = 25,841		*n* = 29,632		*n* = 23,861	
	*n*	%	*n*	%	*n*	%	*n*	%	*n*	%
EDOR, mean (SD)	0.57	(0.25)	0.25	(0.08)	0.46	(0.05)	0.65	(0.06)	0.91	(0.12)
Age, years, mean (SD)	61.8	(21.8)	61.9	(21.8)	61.4	(22.0)	61.7	(21.8)	62.3	(21.3)
Male sex	48,922	47.4	11,516	48.3	12,338	47.7	13,908	46.9	11,160	46.8
Shift										
Day	49,811	48.3	11,551	48.4	13,536	52.4	14,211	48.0	10,513	44.1
Evening	36,556	35.4	1846	7.7	7816	30.2	13,811	46.6	13,083	54.8
Night	16,829	16.3	10,465	43.9	4489	17.4	1610	5.4	265	1.1
EMS transport	57,227	55.5	13,885	58.2	14,789	57.2	16,238	54.8	12,315	51.6
ESI	102,989									
ESI1	9	0.0	2	0.0	1	0.0	3	0.0	3	0.0
ESI2	6565	6.4	1558	6.5	1642	6.4	1957	6.6	1408	5.9
ESI3	93,261	90.6	21,360	89.7	23,290	90.3	26,777	90.6	21,834	91.7
ESI4	2973	2.9	844	3.5	808	3.1	784	2.7	537	2.3
ESI5	181	0.2	53	0.2	63	0.2	43	0.1	22	0.1
Hospital admission	57,746	56.0	12,273	51.4	14,402	55.7	17,038	57.5	14,033	58.8

*EDOR* Emergency Department occupancy ratio, *EMS* emergency medical services, *ESI* emergency severity index

Unadjusted logistic regression analyses showed no significant association between crowding at arrival (EDOR_A_) and patient mortality (Q4 vs. Q1 OR 1.13 [0.94–1.36] *p* = 0.182). The mortality rate slightly increased in Q4 compared to Q1 (1.1% vs. 0.9%), but this result was not statistically significant (*p* = 0.284). After adjusting for confounding factors in the logistic regression analyses, the most crowded quartile (EDOR_A_ > 0.75) was associated with 10-day mortality (OR 1.31 [1.07–1.61] *p* = 0.009) (Table 2). In further analysis the actual threshold value for increased mortality was found to be when the occupancy exceeded EDOR_A_ 0.89 (OR 1.40 [1.05–1.86], *p* = 0.024).

**Table 2:** 10-Day mortality risk for EDOR at arrival, adjusted logistic regression

	OR	95% CI	*p* value
EDOR_A_			
Q1 (< 0.36)	1		
Q2 (0.36–0.54)	1.18	0.98–1.43	0.079
Q3 (0.55–0.75)	1.09	0.90–1.33	0.386
Q4 (> 0.75)	1.31	1.07–1.61	0.009
Age (per year)	1.06	1.05–1.07	< 0.001
Male sex	1.65	1.45–1.87	< 0.001
Shift			
Day	1		
Evening	0.80	0.69–0.92	0.002
Night	0.94	0.77–1.14	0.538
Triage acuity			
ESI 4–5	1		
ESI 3	1.56	0.88–2.78	0.128
ESI 1–2	3.47	1.91–6.31	< 0.001
EMS transport	3.76	3.10–4.56	< 0.001

For abbreviations, see Table 1

When crowding was defined using EDOR_MAX_, the results were largely consistent with those obtained using EDOR_A_. However, in case of EDOR_MAX_, also the unadjusted analyses showed a statistically significant increase in 10-day mortality (Q4 1.1% compared to Q1 0.9%, *p* = 0.009, OR 1.34 [1.12–1.61], *p* = 0.001). After adjusting for confounding factors, the OR for 10-day mortality was 1.27 (1.04–1.56, *p* = 0.020) (See Online Resource 1, Table 1). In the additional analysis performed using EDOR_MAX_, the threshold value for significant crowding was found to be the same as in EDOR_A_, namely, 0.89 (OR 1.38 [1.02–1.86], *p* = 0.035). Higher occupancies (EDOR_MAX_ > 0.97) did not result in higher mortality and were statistically non-significant.

In the multivariable analyses, age, male gender, arriving by EMS, and a higher ESI score were also associated with increased mortality. The evening shift was associated with lower mortality compared to the morning shift (OR 0.80, *p* = 0.002), whereas the night shift did not differ from the morning shift (OR 0.94, *p* = 0.538) (Table 2).

In the subgroup analyses, the odds ratio for 10-day mortality for the admitted patients (*N* = 57,650) was 1.23 (1.00–1.52) in Q4 (*p* = 0.064). Within the discharged patient group (*N* = 45,308) the most crowded quartile showed an OR of 1.79 (0.93–3.46, *p* = 0.084). Although there was an increasing trend in mortality among the discharged patients in each EDOR (at arrival) quartile (Q2 1.36, Q3 1.56 and Q4 1.79), these findings were not statistically significant (Table 3). When using the maximum EDOR to measure the crowding, these results were likewise non-significant (See Online Resource 1, Table 2).

**Table 3:** Mortality among admitted and discharged patients, crowding defined as EDOR at arrival

EDOR_A_	OR	95% CI	*p* value
Admitted (a)			
Q1 (< 0.36)	1		
Q2 (0.36–0.54)	1.16	0.95–1.41	0.148
Q3 (0.55–0.75)	1.03	0.83–1.26	0.804
Q4 (> 0.75)	1.23	0.99–1.52	0.064
Discharged (a)			
Q1 (< 0.36)	1		
Q2 (0.36–0.54)	1.36	0.74–2.50	0.330
Q3 (0.55–0.75)	1.56	0.83–2.90	0.165
Q4 (> 0.75)	1.79	0.93–3.46	0.084

^a^All analyses adjusted with age, sex, shift, ESI, and transport mode

For abbreviations see Table 1

## Discussion

In this study, crowding was associated with increased 10-day mortality in a Nordic combined ED. This includes both admitted and discharged patients as described earlier. In previous studies, the short-time mortality risk ratio for admitted patients was similar varying between 1.05 and 1.34 [8, 9, 11]. However, direct comparisons are difficult due to marked heterogeneity in the way crowding has been defined in different studies. For example, the most crowded quartile can possibly be significantly more crowded in one ED than in another. Of all the studies showing positive correlation between crowding and mortality, only Jo et al. and Berg et al. used EDOR to measure the crowding, and the mean EDORs in their quartiles were markedly higher than ours were [7, 10].

To enable comparison between previous studies, we divided crowding statistics to four quartiles, as has been frequently done in the past [7, 9, 10, 12, 13]. In addition, the actual mortality-associated threshold was sought in further analyses. In our study, this threshold value revealed to be as high as 90% occupancy. The same threshold was found both in EDOR_A_ and EDOR_MAX_.

We studied two different EDOR variables, namely, the occupancy at arrival and the highest occupancy during a patient’s stay. The most crowded quartile showed an increase in patient mortality in both analyses. The difference between these variables was, that if the state of crowding was evaluated at patient arrival, the unadjusted analyses did not show an association between crowding and mortality.

After splitting the data in two to study the admitted and discharged patients separately, the results did not reach statistical significance (Q4 OR 1.23, *p* = 0.064 and 1.79, *p* = 0.084, respectively), even though the odds ratio for 10-day mortality among discharged patients increased linearly in each EDOR quartile. Otherwise, male gender and increasing age were associated with mortality in all ED occupancy states, but the important question for which patients are at the greatest risk, particularly during crowded hours, remains unanswered.

In this study, we were able to use a very precise patient-specific definition for crowding. This has also been the most recommended way of measuring crowding based on earlier studies [15, 16, 20]. The occupancy ratio is a useful metric, since it can be calculated in real-time and thus can be used to assess the level of crowding for clinical purposes, like calling for more staff in time for rush hours. Defining the highest occupancy during a patient’s ED visit is a new and valuable variable to use to describe the effect of excess ED crowding on each individual patient.

This study focused on non-critical ED patients, who require monitoring but not immediate attention in the resuscitation room, as determined by the triage evaluation. We focused on this group as they are particularly vulnerable to the negative effects of crowding when resources are limited, and critical patients must take priority. This group comprises a significant proportion of ED patients, and many may suffer from serious conditions, such as sepsis or acute myocardial infarction (AMI), that require urgent treatment to achieve optimal outcomes.

Even though the association between crowding and patient short-term mortality has been documented in many previous studies, the underlying reasons remain mainly unexplained. Some studies have shown that in crowded situations, for example, the door to needle-time for AMI patients is increased or that antibiotics for septic patients are given later [21, 22]. These kind of delays in initiating vital treatments can play a role in increasing the mortality but are probably not enough to explain it thoroughly. Since the crowding in EDs is often created by congestion in other parts of hospitals, other possible cause for increased mortality can be found in patients boarding in the ED, waiting for inpatient beds [1, 23]. This has been shown to lower the quality for care causing mortality, adverse events and missed medication [24–26]. As a third possible cause, physicians working in the crowded EDs are naturally under greater pressure to discharge the patient and make hasty decisions. The effect of this to patient safety has not been studied as far as we know but is reasonable to be taken in discussion.

## Limitations

The strength of this study is the large study population and comprehensive data gathered on patients’ later outcomes. In Finland, EDs are organized by public healthcare services, meaning that all patients entering the ED were included in the sample, regardless of their private health insurance status. However, the study also had some limitations. This was a retrospective, observational single-centre study conducted in the ED of a single university hospital in Finland, which decreases the generalizability of the results to other EDs.

The most critically ill patients were left outside this study, which is a limitation when comparing the results with other studies. However, we believe this choice to be justified, since these patients tend to get the necessary attention regardless of the crowding status.

All patients visiting the ED are placed in four different treatment spaces based on the severity of a patient’s condition and the suspected cause of illness. Sometimes the patient’s condition deteriorates during the visit and, if necessary, he/she is moved to another space for closer surveillance, yet only the last occupied treatment space is saved in the database.

Many important confounding factors were included in the analyses. However, there were some potential confounding factors that we were unable to adjust for due to unavailable data, such as staff deficits during shifts, patients’ comorbidities, or the prior decision to *Do Not Attempt Resuscitation* (DNAR).

Mortality is a widely used and important clinical endpoint but provides only partial insight into potential adverse effects of crowding. In addition to increased mortality, crowding may have other negative effects on patient outcomes like longer in-hospital stay or delays in treatment, as has been reported earlier [11–13, 21, 22]. The psychological effects of crowding should not be neglected either, as it can lead to increased stress and dissatisfaction among both patients and staff.

## Conclusion

In this study, visiting the ED during the most crowded hours increased the risk for 10-day mortality. Future research should investigate patient-related risk factors that may contribute to increased mortality during crowded periods. This study did not include the most critically ill patients and, in the future, the effects of crowding on this subgroup should be also studied.

### Supplementary Information

Below is the link to the electronic supplementary material.

Supplementary file1 (PDF 879 KB)

## Data Availability

The data that support the findings of this study are available from Well-being services county of Pirkanmaa, Registry at kirjaamo@pirha.fi, but restrictions apply to the availability of these data, which were used under license for the current study, and so are not publicly available. Data are, however, available from the authors upon reasonable request and with permission of Well-being services county of Pirkanmaa.
