# Supplementary material for: Emergency department crowding increases 10-day mortality for non-critical patients: a retrospective observational study
Source: Intern Emerg Med. 2023 Aug 22;19(1):175–81. doi: 10.1007/s11739-023-03392-8 (PMC10827824; doi:10.1007/s11739-023-03392-8)
Supplement: Supplementary file 1 — Supplementary file1 (PDF 879 KB) [file 11739_2023_3392_MOESM1_ESM.pdf]

Online Resource 1

Emergency Department crowding increases 10-day mortality for non-critical patients: a retrospective observational study

Internal and Emergency Medicine

Anna Eidstø[1], Jari Ylä-Mattila, Jalmari Tuominen, Heini Huhtala, Ari Palomäki, Teemu Koivistoinen

[1] Emergency Department, Tampere University Hospital, P.O. Box 2000, FI-33521 Tampere, Finland
Email address: anna.eidsto@fimnet.fi

Table 1. 10-day mortality risk for maximum EDOR, adjusted

	**OR**	**95% CI**	**p-value**
$EDOR_{MAX}$			
Q1 (< 0.56)	1		
Q2 (0.56-0.75)	1.14	0.93-1.38	0.202
Q3 (0.76-0.92)	1.05	0.86-1.29	0.631
Q4 (> 0.92)	1.27	1.04-1.56	0.020
Age (per year)	1.06	1.05-1.07	<0.001
Male sex	1.65	1.45-1.87	<0.001
Shift			
Day	1		
Evening	0.86	0.74-0.99	0.031
Night	0.96	0.78-1.18	0.684
Triage acuity			
ESI 4-5	1		
ESI 3	1.56	0.88-2.77	0.131
ESI 1-2	3.45	1.90-6.27	<0.001
EMS transport	3.77	3.11-4.56	<0.001

EDOR, Emergency Department Occupancy Ratio

EMS, Emergency Medical Services

ESI, Emergency Severity Index

Table 2. Mortality among admitted and discharged patients, crowding defined as EDOR maximum

	$EDOR_{MAX}$	OR	95% CI	p-value
**Admitted**				
	Q1 (< 0.56)	1		
	Q2 (0.56–0.75)	1.04	0.85-1.28	0.705
	Q3 (0.76–0.92)	0.94	0.76-1.17	0.593
	Q4 (> 0.92)	1.12	0.91-1.39	0.287
**Discharged**				
	Q1 (< 0.56)	1		
	Q2 (0.56–0.75)	1.47	0.79-2.76	0.228
	Q3 (0.76–0.92)	1.31	0.67-2.57	0.429
	Q4 (> 0.92)	1.60	0.83-3.10	0.160

* All analyses adjusted with age, sex, shift, ESI and transport mode
For abbreviations see Table 1
